# Supplementary material for: Sirolimus Pharmacokinetics Variability Points to the Relevance of Therapeutic Drug Monitoring in Pediatric Oncology
Source: Pharmaceutics. 2021 Mar 30;13(4):470. doi: 10.3390/pharmaceutics13040470 (PMC8067051; doi:10.3390/pharmaceutics13040470)
Supplement: Supplementary file 1 [file pharmaceutics-13-00470-s001.pdf]

pharmaceutics

MDPI

Supplementary Materials: Sirolimus Pharmacokinetics Variability Points to the Relevance of Therapeutic Drug Monitoring in Pediatric Oncology

GagoAmelia-Naomi Sabo, Sarah Jannier, Guillaume Becker, Jean-Marc Lessinger, Natacha Entz-Werlé and Véronique Kemmel

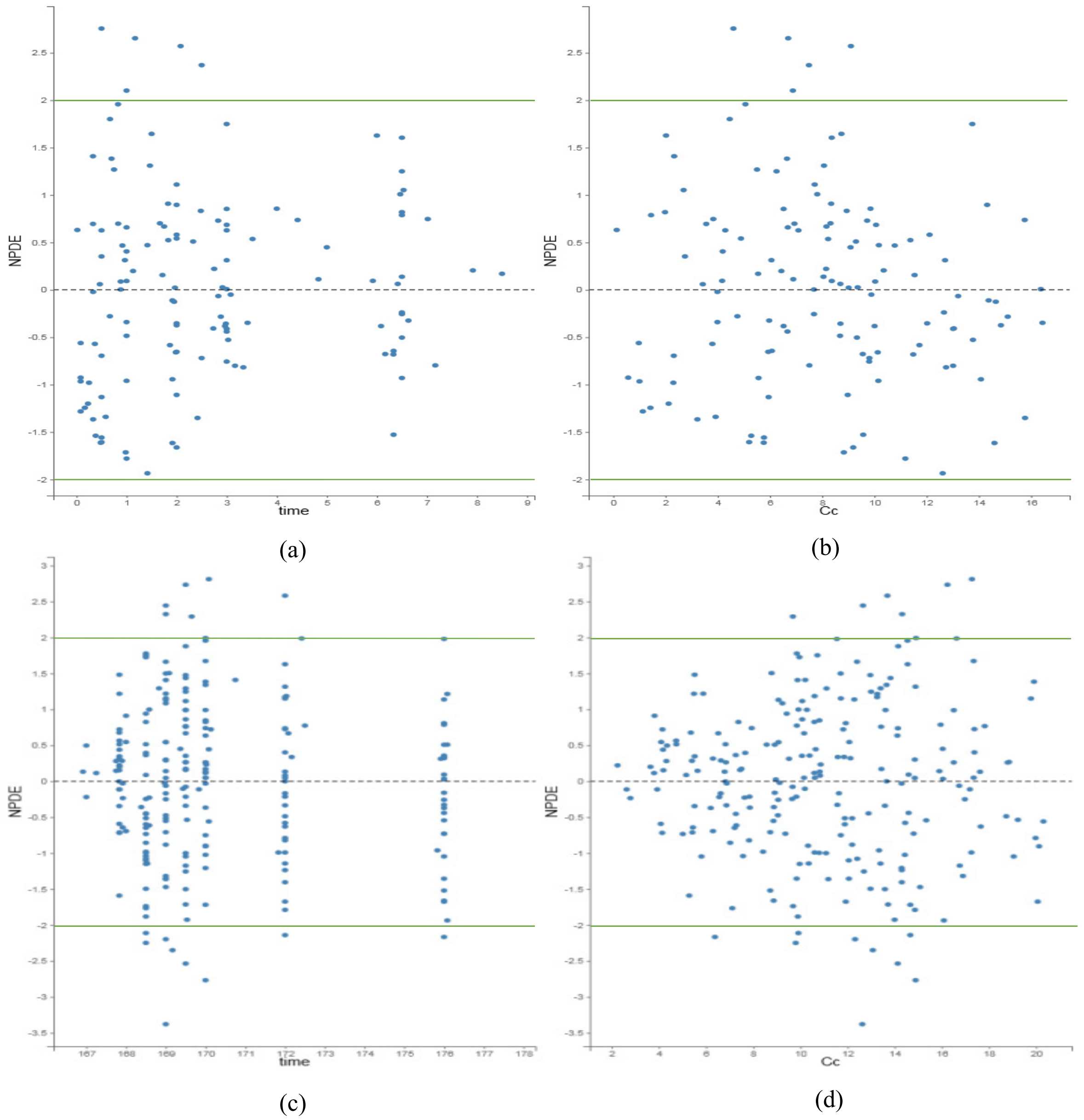

Figure S1. Diagnostic plots. Normalized prediction distribution error (NPDE) *versus* time at D1 (**a**) and D8 (**c**). x-axis: time after first Sirolimus intake (in hours). Normalized prediction distribution error (NPDE) *versus* predicted concentrations at D1 (**b**) and D8 (**d**). x-axis: predicted concentrations (Cc) (in µg/L). Dashed lines represent -2 and +2 interval. Solid line: theoretical NPDE mean.
